# Supplementary material for: Distinguishing excess mutations and increased cell death based on variant allele frequencies
Source: PLoS Comput Biol. 2022 Apr 25;18(4):e1010048. doi: 10.1371/journal.pcbi.1010048 (PMC9071171; doi:10.1371/journal.pcbi.1010048)
Supplement: Attachment — Submitted filename: PLOS CB response.pdf [file pcbi.1010048.s002.pdf]

Dear Editor,

We are submitting the revised version of our manuscript "*Distinguishing excess mutations and increased cell death based on variant allele frequencies*". In the revised version of the manuscript, we have addressed the comments and suggestions of both reviewers and provide a detailed response to each of their points below.

To briefly summarize the major points:

1. We have revised and extended the introduction and the conclusion to improve the presentation of our results and method.
2. We have made extensive clarifications of the details of the mathematical model and its implementation.
3. We have added a simulation study of the robustness of our method to the violation of the assumption that all mutations are neutral that is presented in the Supplementary Information (Fig.S6.) and discussed in the revised version of the main text.
4. We have updated the source code with comments and added inputs for both the simulation and the empirical inferences.

With the highest regards,

Gergely Szöllősi on behalf of the authors

Reviewer's Responses to Questions

Comments to the Authors:

Please note here if the review is uploaded as an attachment.

Reviewer #1: This paper seeks to disentangle the processes of new mutations due to a higher mutation rate or due to higher cell turnover rate. This work assumes a fixed mutation rate and a fixed cell turnover rate during the progression of a tumor, and seeks to estimate these two rates given variant and total read counts of mutations in bulk DNA sequencing data. The authors validate their method on simulated data, finding that while their method is sensitive to per-base sequencing errors, in regimes of low sequencing error the method provides accurate estimates of ground-truth mutation and cell turnover rates. Finally, the paper considers an HCC sample sequenced using a technology with low error, and discusses the estimated rates in light of related findings. While I find the work interesting, I have several comments and questions.

Thank you for the accurate and considered summary.

1. How do cell lineage trees relate to trees inferred using tree deconvolution methods?

The approach we take differs from methods, such as tree deconvolution methods, that attempt to infer complete or partial tumor phylogenies in the crucial aspect that it does not attempt to infer a tree. Rather, our aim is to infer the parameters of an explicit probabilistic model of both mutations and the tree along which they occur by averaging over possible trees. We have revised the introduction to clarify this crucial difference and also discuss tree deconvolution methods:

"Tree deconvolution methods, for instance, attempt to solve this problem by combining phylogenetic inference with a deconvolution step, in which clonal sub-populations from mixed genomic samples are separated prior to or concurrent with inferring phylogenetic relationships between those sub-populations [14,15,16,17].

Our approach also models mutation accumulation along a tumor phylogeny, a cell lineage tree arising from a birth-death process described by the death-to-birth ratio of cells (characteristic of cell turnover). It does not, however, attempt to infer a tree. Rather, it attempts to approximate the approach conceptualized by Felsenstein, wherein the likelihood of a genetic data set is assessed by considering all possible genealogical histories of the data, each in proportion to its probability [18,19]. "

Many clone tree inference methods have been proposed for reconstructing clone trees from bulk DNA sequencing data of tumors. See for example [1,2]. How do these trees differ from the cell lineage trees that you sample in your method? Is there a way to make use of these clone trees in your method?

As described above and in the introduction of the revised version of the manuscript:

"*[..] in contrast to methods such as tree deconvolution and other clone tree methods [15, 16] that attempt to infer complete or partial tumor phylogenies, no single tree is inferred. Instead the parameters of an explicit probabilistic model of both mutations and the tree along which they occur are estimated by approximating the average over all possible trees.*"

2. Copy-number aberrations

VAFs are affected by copy-number aberrations (CNAs). I suggest using cancer cell fractions instead. Also, I would appreciate more details on how VAFs were corrected for CNAs for the HCC data.

As we describe in the methods section of the revised version of the manuscript:

“*We followed the CNV detection steps suggested by the VarScan manual. We used VarScan to obtain raw copy numbers, using the copynumber command. The tumor-to-normal ratio was set to 1.091 for this run. Adjustment for GC content and filtering for minimum region size was done using VarScan's copycaller, with default parameters. Finally, copy number data were smoothed and segmented using the "DNAcopy" circular binary segmentation algorithm in R.*”

3. Justify/discuss assumptions

There are two key assumptions: (1) fixed mutation rate and (2) fixed turnover rate. While the latter assumption is discussed in the Discussion, I would have appreciated simulation experiments that assess how your method would perform if this assumption was violated? Would you still be able to accurately infer the mutation rate?

Lineage-specific changes in the mutation rate or the death-to-birth ratio during the course of tumor evolution will both result in subtrees of the cell lineage tree that are described by different rates and descend from the cell in which the mutation rate or turnover rate changes.

The two changes can be expected to have markedly different effects: a change in mutation rate will from the perspective of the rest of the mutations observed act to “rescale” the branches of the subtree, while a change in death-to-birth ratio will change the shape of the subtree together with the expected number of descendants surviving until the present. A decrease in the death-to-birth ratio (corresponding to an increase in 1-δ, i.e. a positively selected “driver” mutation) is expected to have the most substantial effect on the mutations eventually observed during tumor sequencing.

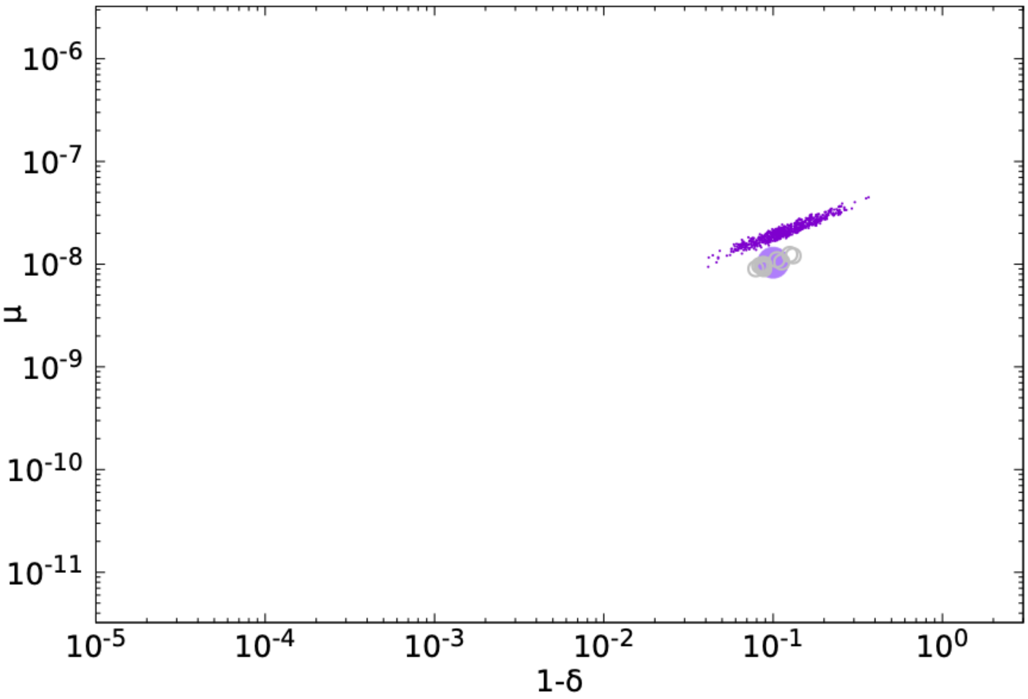

Figure S1. **Robustness to lineage-specific changes in parameters.**

In 500 replicate experiments, we introduced a "driver" mutation that increases 1-δ by 30%. The large purple point indicates the parameter values used to simulate the data, gray circles are inferences for data without driver mutations from Fig. 3 of the manuscript, and purple data points are inferences for data including drivers.

To investigate the robustness of our inference to lineage-specific changes we choose to introduce such "driver" mutations that increase one minus the death-to-birth ratio 1-δ by a substantial 30%. As shown in Figure S6. also reproduced on the previous page, despite the substantial lineage-specific increase the mutation rate is recovered relatively accurately. A small, but systematic increase is apparent in the inferred values of both the mutation rate and the death-to-birth ratio compared to "wild type" values resulting from the subtree with an increased death-to-birth ratio driver lineage.

Moreover, I would like to see a discussion on the assumption of a fixed mutation rate. It has been shown that the mutation rate can change during the evolution of a tumor, due to for instance mutations in DNA mismatch repair mechanisms. Or more generally, due to exposures to mutational signatures. For an example of the former, please see Ref [3].

In addition to the above we also added a discussion of how considering mutational signatures is a promising future direction:

"*At present, we assume a simple uniform mutational process that is independent of genomic context and other mutational processes that shape the genomes of the clones comprising a tumor. In particular, taking into account mutational signatures [33] that incorporate a tumor's evolutionary context [17] based on the cell lineage tree offer the potential to increase both the realism and accuracy of our method.*".

4. More discussion on subclonal mutations clusters.

I found this point extremely interesting. I would appreciate more simulation experiments to further investigate this point. Also, please discuss this in light of the findings in the MOBSTER paper [4].

While we agree that these results are interesting, a more detailed investigation would go beyond the scope of the paper. In particular, as we do not infer a single cell lineage tree, but rather average over trees according to their probability it is not straightforward to design simulation experiments to investigate this question. We have, however, added a discussion of the potential to use our approach as a null model for neutral tumor evolution:

"*The probabilistic model introduced here, together with the associated likelihood calculation and averaging procedure, including the simulation of cell lineage trees, can be extended in a relatively straightforward manner to consider the death-to-birth ratio as well as the mutation rate to be time or lineage dependent or both. Our current model has the potential to act as a null model compared to which models with increased complexity can be compared.*"

5. Extension to support multiple samples

It would be good if the method could support multiple bulk samples from the same tumor.

We agree that modeling spatially resolved mutational diversity of tumors is very promising, as we write in the revised version of the manuscript:

“[..] *currently, the method uses a well-mixed population model for tumor growth and assumes uniform sampling. In the future, a more realistic growth model that includes spatial effects [34, 35] would enhance the applicability of the method opening up the possibility to use data from spatially resolved sampling of tissues, in which the measured mutation frequencies intertwine the correlated ancestry of sampled cells with the prevalence of the mutations.*”

Extending our model to spatially resolved data, however, requires detailed modeling of the spatial growth and dispersal processes that go beyond the scope of the current manuscript.

6. Software and documentation

- Please include example input files.

We now include example inputs.

- Add comments to your code/functions.

We have added comments.

- It looks like your code takes trees as input rather than generating them. Please describe how trees can be generated.

In the “Generating Trees” subsection of the “Appendix” we now write:

“*Cell lineage trees were generated by the ELynx software suite [28], using conditioned birth-death processes. Birth rates, death rates, and tree sizes are freely adjustable with this software.*”

- To facilitate reproducibility of the experimental results, please include simulated data and preferably real data as well.

We now include all data with the code for likelihood calculation at `https://github.com/tg433/evolgenom`

Minor comments:

- turnover or turn over?

Corrected.

- page 3: it'd be good to justify the assumption of independence among sites.

We have added a discussion of this assumption:

“*Similar to other phylogenetic methods we assume that sites collect mutations independently of each other. This assumption is consistent with our assumption of neutral mutations, but is, in general, a simplification that is motivated by computational tractability.*”

- Good to provide the name of the method in the paper.

The current paper is a proof of concept result, aiming to show that the death and mutation rates can be distinguished without inferring a single cell lineage tree. In the future, we plan to develop a more user-friendly, efficient, and general method and would prefer to not name the current exploratory implementation.

- What is the correspondence between the number of mutations and number of leaves in the trees that you sample? More generally, how do you choose the number of leaves?

Our goal was to avoid artifacts in the sampled mutant site data due to the finite size of the tree. Therefore, we chose the number of leaves to be significantly larger than sequencing depth. We found that as long as this criterion was satisfied the results of the inference were robust to the choice of this parameter.

References

[1] El-Kebir M, Oesper L, Acheson-Field H, Raphael BJ. Reconstruction of clonal trees and tumor composition from multi-sample sequencing data. Bioinformatics. 2015;31(12):i62-i70. doi:10.1093/bioinformatics/btv261

[2] Deshwar AG, Vembu S, Yung CK, Jang GH, Stein L, Morris Q. PhyloWGS: Reconstructing subclonal composition and evolution from whole-genome sequencing of tumors. Genome Biol. 2015;16(1):35. doi:10.1186/s13059-015-0602-8

[3] S, Leiserson MDM, El-Kebir M. PhySigs: Phylogenetic Inference of Mutational Signature Dynamics. In: Biocomputing 2020. WORLD SCIENTIFIC; 2019:226-237. doi:10.1142/9789811215636_0021

[4] Caravagna G, Heide T, Williams MJ, et al. Subclonal reconstruction of tumors by using machine learning and population genetics. Nat Genet. 2020;52(9):898-907. doi:10.1038/s41588-020-0675-5

Reviewer #2: In this manuscript Tibély et al. propose a likelihood-based approach to estimate the mutation rate and cell renewal rate history of a tumor from DNA sequencing data from a single bulk sample of the tumor and an adjacent normal sample. The methodology used is sounding, even though the applicability and interest for a wide biological community is questionable and possibly limited due to the strong theoretical character and the various (unavoidable) strong assumptions implied. The method is applied to an experimental dataset, but there is a lack of tools for cross-validation of the inferred parameters with real estimates (even if the method is appropriate for synthetic datasets generated under the same methodological assumptions, which is not striking per se). The mathematical approach is clean but the text suffers from a too technical style that sometimes is more proper of a Technical Note than a Main manuscript. Altogether, in my view the authors should pay most attention on the narrative to better introduce the background and succinctly explain the model's novelty on such a non-new question to be able to convince on its far-reaching biological implications.

Thank you for the positive comments and constructive critique. We have revised and extended both the introduction and the conclusion to improve the presentation.

MAIN POINTS

- The Introduction feels short and too simplistic given the level of concretion of the subsequent approach and it should be more pedagogical to acknowledge the multiple facets and limitations involved in the inference of evolutionary parameters. First, Williams et al. work is referred, but since those authors addressed a similar problem in their original publication and even refined their subsequent analysis based on multiple-sampling data, it is unclear what it was inappropriate in their methodology, or, in other words, what is genuine in this work's approach that makes it that more successful at discerning mutation rate and growth-death ratio even when considering just from bulk sequencing data. Is it just the likelihood approach vs. machine learning methods?

Is it the fact of simulating and fitting the whole VAF distribution rather than its global scaling properties derived from Luria-Delbrück model?

We now elaborate on the novelty of our approach in the introduction:

"*In general, tumor phylogenies represent the evolutionary history of its subclones and can be used to test different hypotheses about tumor evolution. However, the specific features of cancer data pose challenges to the direct application of classical phylogenetic models. In particular, bulk sequencing data contain an unknown number of novel cancer genomes, while classical phylogenetic approaches assume that taxa are known a priori [13]. Tree deconvolution methods, for instance, attempt to solve this problem by combining phylogenetic inference with a deconvolution step, in which clonal subpopulations from mixed genomic samples are separated prior to or concurrent with inferring phylogenetic relationships between those subpopulations [14, 15, 13, 16, 17].*

Our approach also models mutation accumulation along a tumor phylogeny, a cell lineage tree arising from a birth-death process described by death-to-birth ratio of cells (characteristic of cell turnover). It does not, however, attempt to infer a tree. Rather, it attempts to approximate the approach conceptualized by Felsenstein, wherein the likelihood of a genetic data set is assessed by considering all possible genealogical histories of the data, each in proportion to its probability [18, 19]. As a result, in contrast to methods such as tree deconvolution and other clone tree methods [15, 16] that attempt to infer complete or partial tumor phylogenies, no single tree is inferred. Instead, the parameters of an explicit probabilistic model of both mutations and the tree along which they occur are estimated by approximating the average over all possible trees."

Regarding Luria-Delbrück scaling assumptions underlying Williams et al.'s work, we note that, we consider a general birth-death process where birth and death events are independent, in this

case the frequency of rare mutations does not show scaling properties that are invariant with respect to changing death-to-birth ratios.

Secondly, the method shows important limitations (and this is common to Williams et al. work): it assumes a net exponential tumor growth, which is not necessarily realistic, and purely neutral subclonal dynamics. Other authors have shown the prevalence of positive Darwinian selection across tumor types (Martincorena et al, 2017, Cell), and even if this is not incompatible with an excess of neutral passenger mutations, it would affect the subclonal dynamics and the tree shape, consequently affecting inferences from the VAF distribution, as has been largely debated (e.g. Tarabichi et al. 2018 Nat. Genet, as a criticism to Williams et al. 2016). These limitations cannot be ignored and have to be highlighted or authors may want to consider how much inferences would depart from the given estimated values when these assumptions get relaxed. One could draw the impression that estimates can result orders of magnitude deviated if basic evolutionary assumptions are inappropriate. For the same reason, I would avoid overclaims in the Discussion.

We agree that our method shares important limitations with Willimas et al., and clarify this point in the revised version of the manuscript:

"*A decrease in the death-to-birth ratio (corresponding to an increase in $1-\delta$, i.e. a positively selected "driver" mutation) is expected to have the most substantial effect on the mutations eventually observed during tumor sequencing. As shown in Figure S6, despite a substantial lineage-specific increase the mutation rate is recovered relatively accurately. A systematic increase is, however, apparent in the inferred values of both the mutation rate and the death-to-birth ratio compared to "wild type" values resulting from the subtree with an increased death-to-birth ratio driver lineage. This highlights the limitations [35] of the assumption of only neutral mutations, which our model shares with Williams et al. [2] and which is incompatible with recent empirical results on patterns of selection in cancer and somatic tissues [34].*"

- The model definition is not sufficiently clear or precise. Authors use a lineage tree representation but should explain what methodology they use to generate the simulated trees, i.e. T(delta). Is it by Markov-chain Monte-Carlo? Continuous or discrete time? What is the initialization condition and end time? What are the random events?

As we clarify in the model section of the revised version of the manuscript, the cell lineage tree is assumed to come from a birth-death process conditioned to have a fixed number of leaves at the time of observation described in refs. [20] and [21] in the revised version of the manuscript:

"To model the descents of the extant cells, we consider a birth-death process conditioned to have a fixed number of observed lineages (in our case sequenced cells) [20, 21]. We consider a birth rate (which is identical to the cell division rate and is also referred to as the cell turnover rate) b, death rate d, and number of sampled cells n. We measure branch lengths in number cell divisions, i.e., birth events. Consequently, the birth rate b can be considered as a scaling constant that sets the unit of time."

Trees are simulated by our own implementation of the method described in [20] and implemented in the R package TreeSim, as we write in the revised version of the manuscript:

"Cell lineage trees are simulated by our own implementation [39] of the point process described in [20], which is more stable for death-to-birth ratios close to unity than the widely used TreeSim implementation [21]."

From the Methods it seems clear that tree topologies and mutagenesis are simulated independent from one another but a higher level of concretion in the main text would be helpful. In particular, there are two major confusing points:- (i) Whether mutagenesis events are circumscribed to the cell division instant (i.e.

internal nodes) or are simulated too all along branches. The text sometimes gives the impression of the former assumption, but at the same time the division rate is taken as unit of time. This is relevant insofar as in one case both parameters are intermingled and mu changes as a result of changes in delta, while in the other they represent independent measures (delta would impact the mutation burden just indirectly, by modulating the total no. of tumor branches, but would not affect its density) and thus parameters might be more easily resolvable? If mu is truly independent it should not depend on b.

We clarify this point in the revised version of the manuscript, where we write:

"When simulating data we first sample random cell lineage trees with continuous-time branch lengths (measured in units of one over the birth rate) from a con- ditioned birth-death process parameterized by the growth rate $1 - \delta$ and the number of surviving lineages n using the point process approach described in [20]. Mutations are subsequently simulated along branches of this tree: For each branch k the number of mutations is drawn independently from a Poisson distribution with parameter corresponding to the product of the branch length l_k and the mutation rate μ, and appears in $n \cdot f_k$ cells of the final population, where f_k is the fraction of cells that descend from branch k (cf. Fig. 1a) in the final population."

- (ii) Related with the previous, how empirical VAF distributions (that are indicative of tumor subclonal composition) can be directly contrasted with a tree whose branches are said to represent individual cells rather than subclones of multiple cells. I would assume a representation in terms of a subclonal tree where branches represent populations of clonal cells would be more readily contrastable with experimental VAF data. It is unclear how the fitting procedure is done if branches are restricted to individual cells and not clades. As a suggestion, I think it would help to improve the explanation on Fig. 1. This shows site frequency spectra but these are difficult to relate with trees on the left as there is no sketch drawing read sites along the branches. And the paragraph describing this connection between site frequency spectrum and tree topology is confusing.

We have revised and extended the section describing the relationship between the site frequency spectrum and the VAF:

"The data available from bulk sequencing are the mutant and wild type read counts at each site. To illustrate how such data can be used to separate the effects of the mutation rate and cell death intensity, consider the variant allele frequency (VAF) spectrum, which can be estimated from read count data. As shown in (Fig. 1a and b) mutation frequencies in the population reflect the branch length distribution of the tumor's cell lineage tree, the leaves of which correspond to the population of cells at the time the sample is taken, and its root is the most recent common ancestor of these cells. Mutation frequencies in the population, however, are not directly observable. What is observed is a random sample of mutant and wild-type read counts per site. The ratio of the observed mutant and wild-type read counts (also called the variant allele frequency) on average corresponds to the fraction of mutant cells in the population, and the total number of reads is determined by sequencing depth.

Changing the death-to-birth ratio modifies the shape of the cell lineage tree by changing the relative lengths of branches closer to and further away from the root and (Fig. 1a), as a result, modifies mutation frequencies in the population (Fig. 1b and c) and the observed variant allele frequencies (i.e. changes the shape of the variant allele frequency spectrum or VAF, Fig. 1d). Changing the mutation rate, on the other hand, does not have any effect on the branch length distribution as it changes the expected number of mutations uniformly along the tree. Thus, changing the mutation rate leaves the relative frequencies of mutations in the population on average unchanged and, aside from the random sampling of reads, only changes the VAF by on average a multiplicative scaling factor (cf. Fig. 1c and d and Fig. S1)."

On the same line, Fig. 1 would benefit much if it shows trees generated under four different scenarios (low mu-low delta, high mu-low delta, low mu-high delta, and high mu-high delta) or at least under scenarios where just one or the other parameter is changed but not both at the same time like is done here. That would better illustrate how the tree shape and mutation burden change with the two parameters of interest.

Mutation rate only influences the total number of mutations in the site frequency spectra and by extension the VAF, leaving the overall shape of the site frequency spectrum intact and by extension the variant allele frequency spectrum unchanged up to differences due to random sampling of reads. We have added text to clarify this point. We have also added a supplementary figure reproduced below that illustrates the effect of changing the mutation rate.

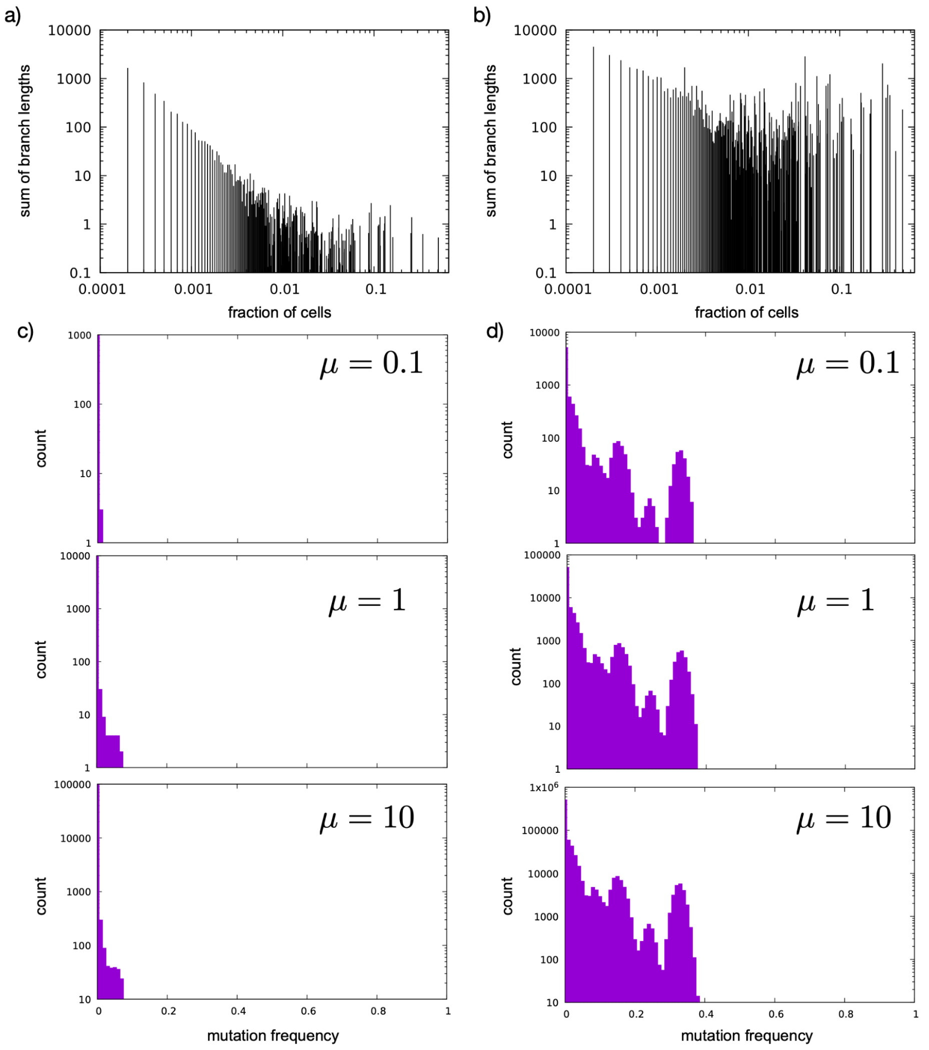

Figure S1. **VAF spectra for varying mutation rates and death-to-birth ratios.**
Branch length distributions for a) low death rate and b) high death rate and corresponding VAFs, respectively c) and d), with the mutation rate varying over two orders of magnitude.

Altogether, the model section is vague and contrasts with that of the parameter estimation procedure which is much more detailed (Indeed, the parameter estimation section is already initiated by the second last paragraph of pg. 3 even if the title comes later).

We have revised the model section, but prefer to present the likelihood of observed data in the model section, as opposed to the parameter inference section, as we think that for the generative model the likelihood is determined directly by the model.

MINOR POINTS

- I in part differ from the diagnosis that the main limitation for evolutionary inference is that bulk sequencing does not resolve the genotypes of individual cells. I assume it is not as much a problem of spatial resolution as it is of temporal resolution, as it is limited to a snapshot. I think exploring models accounting for sampling bulk data at various serial time points is a suitable direction for future work. This is poorly explored. On the contrary, I am not persuaded of what the benefit of sampling individual cell genotypes is vs. sampling subclones in regard to tree reconstruction and evolutionary inferences. This is perhaps a misinterpretation of what individual cell trees in this work offer vs. subclone-based approaches in Williams et al, or they are essentially similarly informative.

As also noted above, our focus is on inferring parameters of describing tumor evolution and agree that time-resolved sequence data is potentially highly informative. This topic, however, is outside the scope of the current work.

- In the Abstract the sentence referring to the "orders of magnitude" difference between mutation burden in tumors and healthy tissues should be toned down.

We have revised the abstract.

It is certainly the case in liver but not necessarily that dramatic in other tissues (authors may want to refer to recent literature by P. Campbell group and colleagues). In any case it deviates the attention from the main purpose of the manuscript that is not on this comparison. This difference in mutation rate is perhaps too much overstated too in pg. 10 given that methods to estimate mutation rate in healthy tissues are so relaxed and heterogeneous. Yet, authors should consider that most reliable method to infer mutation rate in healthy tissues is by fitting data from individuals of different age, and for liver in particular, they can refer to the estimate in Brunner et al (2019) Nature. Here again it is patent that the particular election to normalize mutation rate by cell division is inconvenient as it heavily relies on that estimate, while Brunner et al. present a mutation rate as an independent parameter with dimension site-1 real time-1.

We have added a reference to Brunner et al.

- Fig. 3 is very nice, even if it is limited to some particular true mu:(1-delta) pairs. I would suggest an accompanying figure (e.g. a heatmap), but not necessarily a comprehensive one, showing how uncertainty in parameter estimates changes across a grid search on different true values of mu and (1-delta) extending to other regions of the grid. Do changes in mu affect uncertainty for the same value of delta?

We have explored possibilities for representing the data presented in Fig.3 as a heatmap but found that it would require a significant amount of additional computation, while only adding a limited amount of clarity to the presentation.

It is certainly interesting how uncertainty increases when the death rate approaches the growth rate. The authors point to the fluctuation on the shapes of the trees used for sample generation. I think this deserves a brief reflection. If I am right, I understand that the difficulty in capturing the distribution of bifurcation times with limited leave sized trees in cases where d is similar to b arises from the broad distribution of possible outcomes when fates are balanced in stochastic branching birth-death processes (Bailey, 1964, The elements of stochastic processes with application to the natural sciences). Similar problems arise in healthy tissues (Snippert et al, 2010, Cell; Piedrafita et al, 2020, Nat Commun). Indeed, when commenting on possible reasons for tumors to show elevated values of delta, the underlying tissue architecture and stem cell renewal mode might play important roles too determining the tumor death-to-birth ratios. These factors can be reflected too in the discussion in pg. 11. For instance, the organization of intestinal epithelial stem cells into crypts imposes constraints on clonal expansion between crypts while there is intrinsically elevated competition forces between stem cells belonging to the same crypt (Snippert et al, 2010, Cell).

We extended the discussion to include this point.

- The considerations when discussing the cell division rate of the HCC tumor are pertinent but the authors neglect other major factors, such as the significant fraction of the HCC volume occupied by cirrhotic tissue and stroma, or the heterogeneous dynamics between tumor cells depending on their genotype (Darwinian selection) and/or the microenvironment that can greatly affect those estimates. They should tone down the argument that “division rate is realistic, suggesting that the approximation is adequate” – they should do so too when arguing that the fact that the value falls unrealistic when considering random sampling from a large specimen suggests that the sampling ratio may be close to 1.

Done.

- I would suggest to change the title for: “Distinguishing excess mutations and increased cell renewal based on tumoral variant allele frequencies”, which is more understandable.

We would prefer not to add “tumoral”, because, while the primary application we consider is tumor sequence data, the method is more general.

- Regarding the mathematical formulation, the two conditions for m reflected in Eq. 6: is it the intersection between the two sets that is considered? In Eq. 7, better to include a parenthesis for readability, to show that second summation term is nested to the first.

Done.

Both in Eq. 6 and 7 subindexes i might be pertinent for r_i, m_i, m’_i and m_th,i.

We have on purpose neglected the site indices, as we believe that they hinder comprehensibility.

- As a suggestion, variable names m_th, m’, M_obs mut can be explicitly mentioned in the text right before their first use in the respective equations.

Done.

- Eq. 7 could benefit from a little explanation in the accompanying text saying that it is a way of weighting the influence of coverage on the discovery of any actual given branch length from just significant mutant read counts, which should thus be < or = to Sum_k l_k. In this sense, I wonder how much this consideration affects the estimates in practice? Can some values for cases where mu is estimated ignoring the significance criterion be represented in Fig. 3? Similarly, in the same paragraph, authors may want to explain that Msig is similar to the total number of accumulated true mutations.

The threshold in mutant counts is important for non-zero sequencing errors, and neglecting it significantly impacts accuracy in such cases.

- The definition and extent of a "synthetic dataset" is unprecise. I infer that when "10 synthetic datasets were generated for each true value" it means that 10 query trees were generated for the same given point in the delta grid search, and then "10^4 trees with 10^4 leaves were used for fitting" it means per each dataset?

Corrected.

- Similarly, mu is said to be estimated directly from the data, but since mu_est requires L_sig computation and this depends on T(delta), one infers that it cannot be uncoupled from the delta inference; in other words it is not estimated beforehand but in conjunction with the likelihood for delta, for being a related quantity. Is this correct? This reflection would clarify the procedure.

Yes, the estimate of the mutation rate depends on T(delta), corrected.

- What is the criterion when selecting a particular number of simulated trees (10^4)? Is this based on empirical observation, e.g. an optimality criterion like the value for which the error on the estimate gets below a certain threshold or similar?

Regarding the choice of 10^4 leaves, it seems to fulfill the number of cells in the empirical HCC dataset, but I wonder if fitting bulk data from a larger tumor sample would require more tree leaves or not necessarily insofar as leaves are representative of the overall subclonal heterogeneity? i.e. Can we assume one branch = one subclone in the formalism used?

We found that as long as a sufficiently large number of leaves is used, estimates are robust to this choice.

- Confidence intervals on MLE values should be provided for the inferred parameters, at least for the empirical dataset. From pg. 14 it is unclear whether a confidence interval is implemented or not but authors could resort to a Likelihood Ratio test or similar in the light of supplementary figures.

Due to the averaging procedure used cannot be formally interpreted as a likelihood and in practice, we found that likelihood-based tests give unrealistically tight confidence intervals.

- I am just curious to know why not defining delta as a birth-death ratio instead of death-birth ratio. In any case, the election to represent one minus death-to-birth ratio is already sufficiently twisted so that figures might benefit from indicating the extremes corresponding to 0 death and max. death.

We now explain that

“We only consider exponentially growing cell populations (i.e., $\delta < 1$), the growth rates of which in units of cell birth are $(b - d)/b = 1 - \delta$.”

And we have extended the legend of each figure to clarify the change of the death rate along the axes.

- When introducing the likelihood model, it would be useful to state what exactly “observed data D” is. Indeed, is “site frequency spectrum” equivalent to “distribution of variant read counts”? I find less confusing the latter term

Corrected.

- Authors may acknowledge in pg. 9 if empirical data corresponds with WGS or WES, and whether it involved amplification that could impair the interpretation of the VAF distribution.

In the revised version of the manuscript we explicate that the data used is a partial WGS sample.

TYPOS AND REWORDING

- multiple sites where the term “linage” is used and it should read “lineage”

Corrected

- (pg. 2) “mutations from individual cells are intermixed” -> rephrase

Corrected

- (pg. 2) “with the cell linage tree” -> “in terms of a cell lineage tree”

Corrected

- (pg. 3) “descendance of the extant cells” -> “descendance of any given cell”

Revised.

- (pg. 9) Perhaps one could simply point to (ii) the level of randomness on tree structure and (iii) the randomness on mutation occurrence as main sources of noise apart from the limited size of trees (i)?

- (pg. 12) “are higher than expected for healthy tissues” -> not true in the case of delta. Rephrase.

Done.

- (pg. 14) “from a prescribed distribution” -> an obscure term… A Poisson?

Revised.

- (pg. 14) “we generate a sample tree” -> “we generate a sample of a given set of simulated trees”

Done.
